# Clinic of the Jefferson Medical College

**Published:** 1852-03

**Authors:** O. A. Judson

**Affiliations:** Clinical Clerk


### CLINICAL REPORTS.

*Clinic of the Jefferson Medical College. Service of* PROFESSOR DUNGLISON.

(Reported by O. A. Judson, M. D., Clinical Clerk.)

*Clinic of January 14th*, 1852.

*Cases presented*:—Chronic Bronchitis; Rheumatism; Tuberculosis of the Lungs, 2 cases; Psoriasis inveterata; Acute Bronchitis; Lupus non exedens; Arthrodynia; Chronic Laryngitis; Lithuria; Lichen; Lepra; Pityriasis; Torpor of Colon, 2 cases; Tænia solium; Tophaceous concretions in the joints; Favus; Diabetes Mellitus; Emesis; Faulty innervation of heart.—Total 22.

CASE 1.—George L., æt. 25, by occupation an umbrella-maker, presented himself this day, having suffered for some time from cough and slight dyspnœa. Various anodyne mixtures were prescribed for his relief, but thus far without any appreciable benefit. His general appearance is that of a person laboring under pulmonary disease; countenance pallid and sunken; eyes having a peculiar shining lustre; has had hæmoptysis and daily exacerbations of fever, but no colliquative sweats. On inspection, the right side of the thorax is observed to rise more than the left, during the act of inspiration, which is rendered more evident by palpation of the anterior walls of the chest during the respiratory process. Percussion exhibits a decided dulness in the infraclavicular region of the left side, with absence of respiratory murmur, with tubal respiration, and bronchophony. The exaggerated respiration in the right lung gives evidence that the great burden of the function is thrown upon it.

The inference drawn from the combination of the rational and physical signs was, that the patient labored under a tubercular deposition at the summit of the left lung; which however gave no signs of having proceeded to extensive softening.

The indication for the treatment of this case is plain; to modify the whole system of nutrition. This can only be done by modifying the condition of the fluid of the circulation, so that it may exert a new impression upon the tissues through which it is distributed, and thus induce a new action in them. Experience has shown that nothing is better calculated to fulfil this indication than an animal oil, such as the oleum morrhuæ. This oil probably acts altogether dietetically, producing the same deposition of fat as follows the use of sugar when taken largely. The relative value of the different animal oils has not been rigorously determined; but there is reason for the belief, that the cod-liver oil does not possess any eminent advantage over other animal oils; and that similar results follow the use of vegetable oils. In order to test the effects in such cases of another animal oil, the common sperm oil has been purified by the apothecaries of the College by animal charcoal; and the class will observe, that its taste and smell are not much more, if at all more, objectionable than those of the oleum morrhuæ. It is this oil that is meant when the *oleum cetaceum* is prescribed in this clinic. The patient will take a dessert-spoonful three times a day in hot coffee; and, to improve the condition of the fibrinous element of the blood, which appears to be badly elaborated in tuberculosis, he will use animal food freely; relieving the cough by any of the ordinary demulcent and opiated expectorants.

Case 2.—The next patient presented was Francis H., æt. 21, by occupation a stone mason. He had complained for a considerable time of a dull aching pain, which he referred to the region of the kidneys, and which would occasionally shoot down towards the bladder, along the course of the ureter, causing him great suffering. His urine was subsequently examined, and found to be acid, and to deposit urate of ammonia. He was put upon an alkaline treatment, fifteen grains of bicarbonate of soda three times a day, from which, (January 21st), he has experienced considerable relief.

Case 3.—The attention of the class was next directed to a case of glucosuria or diabetes mellitus. John S. V., æt. 36, an operative in one of the manufactories of Chester county, has been sent to Dr. D. by a respectable practitioner under whose care he had been for some time, and who had accurately appreciated the morbid condition.

About twelve months since, he perceived, that his flesh and strength were beginning to fail, and shortly afterwards he noticed an increased discharge of urine of a sweet taste, which, during the last three months, has become quite profuse, amounting, at times, to fourteen pints *per diem*. It is now, however, reduced to six or eight; yet he is still obliged to rise two or three times during the night to evacuate his bladder. From the outset of the disease his appetite has been voracious; thirst insatiable. His physician put him on the use of five grains of the pulvis ipecacuanhæ et opii, and of four drops of creasote, three times daily. He thinks, however, that his improvement was coincident with his being placed upon an exclusively animal diet.

The urine was subjected to different tests, and the results exhibited to the class; first, to the test of Trommer, which consists in adding to the suspected urine a small quantity of a solution of sulphate of copper, merely enough to give a blue tint,—phosphate of copper is precipitated. Liquor potassæ is then added in excess,—and the hydrated oxide of copper is thrown down, which soon dissolves in the alkaline solution, leaving the liquid of a blue color resembling ammoniuretted copper. It is then heated to ebullition, and if sugar be present the solution yields a precipitate of the red oxide of copper.

This test was perfectly satisfactory as was shown by the specimens on the table.

The test of Moore was also applied and with similar results. This consists, in adding liquor potassæ to the urine and boiling for a few moments, when it assumes a brown color, the intensity of which is proportionate to the amount of sugar present.

Another, and the best test is the addition of a little yeast, and subjecting the urine to a gentle heat; when the supervention of the vinous fermentation, will show conclusively the presence of saccharine matter. Such was the case here.

However profuse the secretion of urine may be in glucosuria its specific gravity is always greater than in the healthy condition. In this instance it is 1029. It is often much higher than this, but as a general rule it may be said to vary from 1020 to 1050. On weighing the extract obtained by evaporation, it will be found to hold a direct proportion to the amount of urine passed. Based upon this fact, a table was prepared by Dr. Prout, and modified by Dr. Golding Bird, by which we may arrive at an approximation of the amount of sugar passed, without going through the operose process of evaporation. For example : if the specific gravity be 1029, the quantity of solid matter in a pint, according to the table, will be 555.2 grains. By means of such calculations, we are enabled to say how much saccharine matter is secreted from the blood during any given period. For if 555.2 grains of solid matter be contained in every pint, and as many as eight pints are voided during the day, we know that 4441.6 grains of sugar will be passed *per diem*, and such is the quantity discharged by the patient at this time.

The different modes of taking the specific gravity of urine were then explained by means of the areometer or urinometer, and of the specific gravity bottles of 1000 grains and 100 grains respectively, all of which were exhibited, as well as the more operose but always available method with an ordinary vial.

The precise nature of the morbid condition in glucosuria is not manifest. A great variety of opinions has prevailed on the subject, and it still remains an unsettled point. Necroscopic examination has aided but little in the inquiry. The kidneys, have at times presented a hyperæmic condition, with occasionally hypertrophy, which might be anticipated from the incessant duties entailed upon them. For a long time, indeed, it was maintained that the disease must be referred to those great depurating organs ; but subsequently more attention was directed to the condition of the blood, which many modern pathologists have considered to be primarily affected. In the progress of well devised and well conducted experiments, unequivocal signs of the presence of sugar have been found in the serum of the blood, and of late the presence of sugar in that fluid has been confirmed by distinguished chemical observers : and to account for its presence there, it has been maintained by many, that the fault is in the apparatus of primary assimilation—that sugar is formed in the stomach during the digestive process, and that, consequently, our remedial measures should be addressed to the deranged condition of the digestive apparatus.

Two distinct views, in this relation, have been maintained by MM. Bouchardat and Mialhe. The *former* is of opinion, that the saccharine matter in the blood of a diabetic patient owes its origin to too great a conversion of starch into sugar occurring in the stomach, owing to the generation there of a principle analogous to the *diastase* in barley, which is the active agent in the formation of malt. The *latter*, also, believes in the conversion of starch into sugar—*glucose*—by an *animal diastase* united with it during insalivation. This glucose is transformed under ordinary circumstances by the alkalies in the blood into new products, which are destined for ulterior purposes; but in default of the proper alkaline character of that fluid, the sugar is not transformed; nor is it assimilated, but ultimately thrown out of the economy, and mainly by the kidneys.

Allusion was then made to the recent discovery by M. Bernard of sugar in the liver, and in the blood of the hepatic veins, vena cava and right side of the heart, no matter what may have been the kind of food taken; and to the singular results, that if the medulla oblongata, especially about the floor of the fourth ventricle, or the pneumogastric nerves, be irritated, an increased quantity of sugar is immediately found in the liver, and soon afterwards a large amount in the urine,—phenomena, which, whilst they may not account for the seat of glucosuria being in the pneumogastrics, sufficiently show, that an increased production of hepatic sugar may be intimately connected with the condition of the nervous system.

From the numerous researches which have been made into this interesting, but difficult subject, and taking into consideration all the arguments that have been adduced by different observers, it may be inferred, that in glucosuria, not only the digestive organs, but the whole system of nutrition or second assimilation may be perverted; that instead of the tissues being disintegrated and their elements combined in the usual healthy manner, they may be brought together in the proportions necessary for the formation of saccharine matter, which, by its presence in the blood, acts as an irritant to the kidneys and gives occasion to the increased secretion of urine, whilst the emaciation ensues more particularly, perhaps, from a perverted condition of the cells of assimilation, the manufacture of sugar thus taking place at the expense of the system.

The treatment of diabetes has varied according to the pathological views embraced by observers. Formerly, when its nature was less investigated than at present, it was usual to address the remedies to the kidneys—thus treating the effect, rather than the cause; but at the present day, therapeutists, generally, regard the profuse secretion of urine only as a phenomenon indicating a morbid condition elsewhere, and accordingly direct their efforts to the portion of the system which may seem to them to be mainly concerned in producing the mischief. They who regard the stomach as the organ chiefly in fault, address their principal attention to that viscus. But under the impression that the disease is a vice in the system of nutrition, of the exact nature of which we are ignorant, remedies of a eutrophic character, which may be adapted for modifying the entire system of nutrition, would seem to be indicated. There is no single remedy, however, in the catalogue of the materia medica, that appears to be possessed of much efficacy, and too often the disease proceeds onwards, more or less modified by diet and regimen, but is not the less certainly fatal. Unless an entire revolution can be effected in the nutritive process, no permanent good can be accomplished. Diaphoretics of various kinds have been prescribed in consequence of the dryness of the surface that attends the disease; but this is doubtless owing to the increased urinary depuration, and is consequently only an epiphenomenon. Still it may be well to advise the warm bath and warm clothing, as flannel next the skin, to direct the fluids towards the surface.

On one point in the treatment almost all therapeutists are agreed,—the use of an exclusively animal diet. In fact it were no more than reasonable to conclude, that aliments of ternary composition, consisting of oxygen, hydrogen, and carbon, of which the more purely vegetable matter is composed, should be more readily convertible into sugar, than substances of quaternary composition, that contain also nitrogen, which has to be disposed of. Most unquestionably, the saccharine matter has diminished in quantity under the use of animal food; but it may admit of a question, whether this was not in part owing to the alteréd nutrititive acts occasioned by restricting one, who has been omnivorous or accustomed to both animal and vegetable food, to the former only. The records of the recent voyage from this country to the Arctic regions, in search of Sir John Franklin, will show that when scurvy prevailed to a fearful extent, a slight change in the diet gave rise to a thorough modification in the whole system of nutrition; and fresh animal food obtained by shooting the awk on its first appearance, in its periodical migration, arrested the malady as if by magic. Usually, it has been deemed indispensable for the production of so desirable a result, that fresh vegetables, containing a full supply of the organic acids, should be freely allowed.

In confirmation of the idea, that any thorough change in diet may induce an entire modification in the system of nutrition in glucosuria, it has been found, where the patient, accustomed to a mixed diet, has been restricted to substances of ternary composition—as oils—or to a vegetable regimen, a similar diminution in the quantity of saccharine matter discharged has been observed. Green garden vegetables, as parsley, celery, spinach, are of quaternary composition, and are not liable to the same objections as the amylaceous and saccharine substances.

In consequence of the ready conversion of starch into sugar, Bouchardat recommends what is called "Gluten bread," from which all amylaceous matter has been removed, as an article of diet; but so much disgust is generally caused by it that it can rarely be persevered with.

The general principles laid down are applicable to the case before the class, and will form the foundation of treatment.

Case 4.—Sarah B——, ætat. 8, was next presented. She had labored under a cutaneous affection of the leg for a long time, which has resisted various kinds of treatment. The disease belongs to the class of squamous affections, and falls under the head of Lepra—Lepra vulgaris.

We will place her upon the eutrophic agency of iodine, giving four drops of the liquor iodinii compositus three times daily. As local applications she will have recourse, several times a day, to ablutions with castile soap and water, the object of which is to remove the incrustations, so that the remedial agents may come into immediate contact with the morbid surface; each ablution to be followed by the application of a slightly stimulating unguent, which may consist, in this case, of ten grains of the iodide of sulphur incorporated with an ounce of lard—the affected parts to be kept constantly covered with oil-silk, so as to prevent the incrustations from forming, and to shield the surface from the desiccative and irritating influence of the air. The class would have numerous opportunities for witnessing the excellent effects of this system of treatment in chronic cutaneous diseases.

An instance of these good results was shown in the case of John H., aged —, who had been under treatment since October last for an affection of the hairy scalp,—Trichonosis.

He had been using the liquor potassæ arsenitis in the dose of four drops three times a day, and had applied locally an ointment consisting of equal parts of unguentum picis, ung. sulphuris, and unguentum hydrargyri oxidi rubri. Not much benefit having resulted from this course, he was directed to keep the head closely shaved, to wash it with soap and water six or seven times a day, and afterwards with a solution of a teaspoonful of impure sub-carbonate of potassa (pearl-ash,) in a quart of water, and to keep the head covered night and day with an oil-silk cap.

Having been put for a short time on this treatment, he has returned vastly improved.

